# Peritumoral edema in breast cancer at preoperative MRI: an interpretative study with histopathological review toward understanding tumor microenvironment

**DOI:** 10.1038/s41598-021-92283-z

**Published:** 2021-06-21

**Authors:** Nora Jee-Young Park, Ji Yun Jeong, Ji Young Park, Hye Jung Kim, Chan Sub Park, Jeeyeon Lee, Ho Yong Park, Jin Hyang Jung, Wan Wook Kim, Yee Soo Chae, Soo Jung Lee, Won Hwa Kim

**Affiliations:** 1 Department of Pathology, School of Medicine, Kyungpook National University, Kyungpook National University Chilgok Hospital, Daegu, South Korea; 2 Department of Radiology, School of Medicine, Kyungpook National University, Kyungpook National University Chilgok Hospital, Daegu, South Korea; 3 Department of Surgery, School of Medicine, Kyungpook National University, Kyungpook National University Chilgok Hospital, Daegu, South Korea; 4 Department of Oncology/Hematology, School of Medicine, Kyungpook National University, Kyungpook National University Chilgok Hospital, Daegu, South Korea

**Keywords:** Breast cancer, Cancer microenvironment, Tumour biomarkers

## Abstract

Peritumoral edema (PE) of breast cancer at T2-weighted MR images is considered a poor prognostic sign and may represent the microenvironment surrounding the tumor; however, its histopathological mechanism remains unclear. The purpose of the study was to identify and describe detailed histopathological characteristics associated with PE at preoperative breast MRI in breast cancer patients. This retrospective study included breast cancer patients who had undergone preoperative MRI and surgery between January 2011 and December 2012. Two radiologists determined the presence of PE in consensus based on the signal intensity surrounding the tumor at T2-weighted images. The following detailed histopathological characteristics were reviewed by two breast pathologists using four-tiered grades; lymphovascular invasion, vessel ectasia, stromal fibrosis, growth pattern, and tumor budding. Tumor necrosis and tumor infiltrating lymphocytes were assessed using a percent scale. Baseline clinicopathological characteristics, including age and histologic grade, were collected. The associations between detailed histopathologic characteristics and PE were examined using multivariable logistic regression with odds ratio (OR) calculation. A total of 136 women (median age, 49 ± 9 years) were assessed; among them 34 (25.0%) had PE. After adjustment of baseline clinicopathological characteristics that were significantly associated with PE (age, T stage, N stage, histologic grade, and subtype, all *P*s < 0.05), lymphovascular invasion (*P* = 0.009), vessel ectasia (*P* = 0.021), stromal fibrosis (*P* = 0.024), growth pattern (*P* = 0.036), and tumor necrosis (*P* < 0.001) were also associated with PE. In comparison with patients without PE, patients with PE were more likely to have a higher degree of lymphovascular invasion (OR, 2.9), vessel ectasia (OR, 3.3), stromal fibrosis (OR, 2.5), lesser degree of infiltrative growth pattern (OR, 0.4), and higher portion of tumor necrosis (OR, 1.4). PE of breast cancer at MRI is associated with detailed histopathological characteristics of lymphovascular invasion, vessel ectasia, stromal fibrosis, growth pattern, and tumor necrosis, suggesting a relevance for tumor microenvironment.

## Introduction

Even with advanced screening and treatment strategies, breast cancer is still a significant contributor to cancer-related mortality and shows a substantial variability in the risk of disease progression or therapeutic resistance because of a high grade of intratumoral heterogeneity. To elucidate more individualized prognostication and stratified decision making, various biomarkers have been proposed beyond the traditional prognostic profiles, such as TNM stages. Certain radiomic features in routine medical imaging, including magnetic resonance imaging (MRI), have the potential to serve as non-invasive imaging biomarker that predicts the biologic behavior of the cancer. Breast MRI is being refined as a tool to provide multiparametric information such as morphologic and functional parameters of tumors, as well as diagnosing or staging of breast cancers and monitoring of treatment response.

Peritumoral edema (PE) is seen as a high signal intensity that surrounds the tumor on T2-weighted images at MRI and has been an emerging semantic feature that is related to poor prognosis in breast cancer in current studies^1–4^. However, those findings have been based solely on a phenomenological approach with the lack of pathophysiological explanation, leading to an incomplete understanding of the imaging feature. Even though the preceding studies indicated that PE was associated with some conventional histopathological findings (e.g., pathologic T stage, histologic grade, etc.), they did not explore any detailed histopathological findings and focused only on the tumor itself that overlooked the peritumoral environment. An endeavor to elaborate on the mechanism underlying PE will be ultimately related to the understanding of the tumor microenvironment which is the environment around a tumor^5,6^. There has been a growing interest toward the understanding of tumor microenvironment, and emerging evidences show that the tumor stroma has important roles in tumor initiation, progression, and metastasis^7,8^. Therefore, the purpose of this study was to identify and describe detailed histopathologic characteristics associated with PE at preoperative MRI in breast cancer patients.

## Materials and methods

### Study population

**Figure 1:**
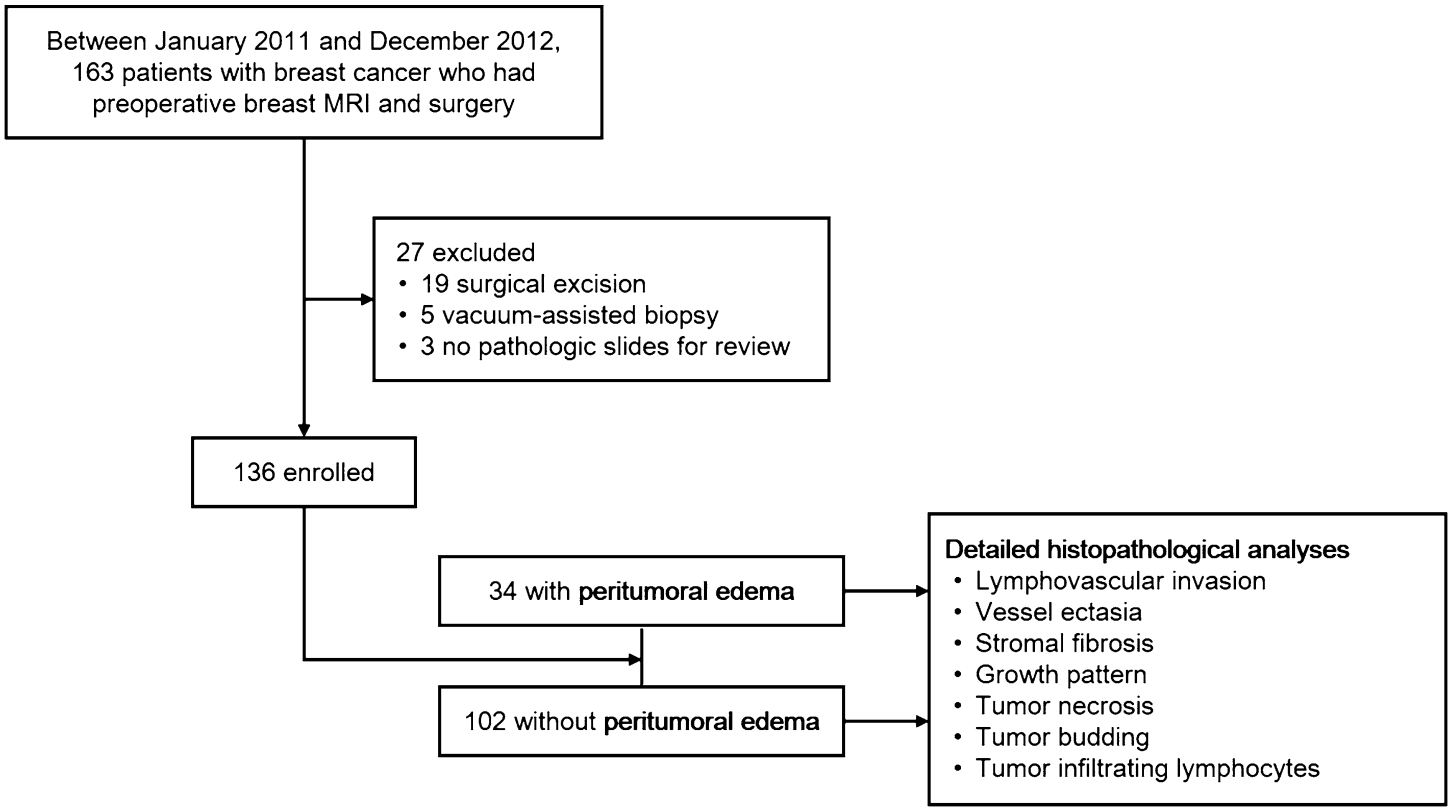
Flowchart shows study enrollment.

Institutional review board (IRB) of Kyungpook National University Chilgok Hospital approved this retrospective study and all methods were carried out in accordance with relevant guidelines and regulations. The requirement of informed consent was waived under the IRB of Kyungpook National University Chilgok Hospital. This study was undertaken within the cohort of 163 women who had a diagnosis of invasive carcinoma of the breast between January 2011 and December 2012, were treated surgically, were not treated with neoadjuvant chemotherapy, and had preoperative breast MRI before surgery (Fig. 1). Study cohort in this study has been shared in our previous study; however, the study purpose and design were different. Whereas the previous study was to investigate prognostic outcome for imaging finding of PE, this study was to focus the microscopic understanding of PE. Furthermore, imaging review for the assessment of PE at MRI and detailed and semi-quantitative histopathological review were specified for the present study. In our institution, breast MRI has been routinely performed for the initial staging in patients with biopsy-confirmed breast cancer. Among them, we excluded women who had undergone surgical excision (n = 19) or vacuum-assisted (n = 5) biopsy prior to the MRI examination and had no pathologic slides for the retrospective review (n = 3). Finally, 136 patients were included in this study.

### MRI acquisition and analysis

The protocol of breast MRI was the same as our previous studies^2,9^. The breast MRI was performed in the prone position using a 3.0-T system (Discovery MR750, GE Healthcare, Waukesha, WI) with a dedicated eight-channel surface breast coil. Axial T1-weighted images (repetition time ms/echo time ms, 746/10; matrix, 352 × 256; slice thickness, 3 mm) and axial fat-suppressed T2-weighted images (8087/88; matrix, 384 × 256; slice thickness, 3 mm) were acquired. Dynamic contrast-enhanced bilateral axial MRI examination included one precontrast and five postcontrast phases using three-dimensional gradient-echo, fat-suppressed, T1-weighted imaging (4/2; matrix, 288 × 416; flip angle, 15°; slice thickness, 1 mm).

Two breast-imaging radiologists (W.H.K. and H.J.K., with 11 years and 19 years of experience, respectively), blinded to the clinicopathological information, assessed the PE based on the degree of the signal intensity surrounding the tumor on T2-weighted images in consensus. PE was defined as the presence of signal intensities surrounding the tumor as high as that of water. Tumors with no or equivocal (moderately high but less than that of water) high signal intensity were classified as negative for PE. In patients with multicentric tumors, only the lesion that has the largest diameter was assessed.

### Baseline clinicopathological analysis

Baseline clinicopathological characteristics, including the age at cancer diagnosis, pathological T and N stages, histologic type, histologic grade, estrogen receptor (ER), progesterone receptor (PR), human epidermal growth factor receptor (HER2) status, and Ki-67 index, were obtained from medical records or surgical histopathology reports. The histologic subtype was based on the World Health Organization (WHO) tumor classification for the breast^10^. The histologic grade was evaluated using the modified Bloom-Richardson histologic grading system. The expression of ER, PR, and HER2 was assessed using immunohistochemical staining. The expression of ER and PR was quantified using the Allred score (a total Allred score > 2 is positive for ER or PR)^11^. Samples with 1–100% of tumor nuclei positive for ER and PR were interpreted as positive^12^.

An HER2 value of 0 or 1 was considered negative (HER2-negative) and a value of 3 was considered positive (HER2-positive). An HER2 value of 2 was considered equivocal; for these cases, silver-enhanced in situ hybridization was carried out and an HER2/chromosome enumeration probe 17 (CEP17) ratio ≥ 2.0 or an HER2/CEP17 ratio < 2.0 with an average HER2 copy number ≥ 6.0 was considered positive (HER2-positive)^13^. A hormone receptor (HR)-positive status was defined as the presence of tumors that expresses ER and/or PR. For the Ki-67 expression status, a nuclear staining of at least 14% indicated a high level of expression^14^. Based on these immunohistochemical staining, tumors were classified into four subtypes: luminal A (HR-positive and low Ki-67 expression), luminal B (HR-positive and high Ki-67 expression or HER2-positive), triple-negative (HR-negative and HER2-negative), and HER2-enriched (HR-negative and HER2-positive).

### Detailed histopathological analysis

Specimens were examined from multiple sections of the tumors and stained with hematoxylin and eosin (H&E). All available specimens were independently scrutinized for detailed histopathological analysis by two pathologists (N.J.P. and J.Y.J., with 10 years and 12 years of experience in breast pathology, respectively) in a blinded manner without information about PE. Discrepant cases were repeatedly reviewed until a consensus was reached. The following characteristics were reviewed using a semi-quantitative, four-tiered grading system (1–4); lymphovascular invasion (LVI), vessel ectasia, stromal fibrosis, and growth pattern.

LVI was defined as a tumor embolus within the vascular channel (lymphatic and/or blood vessels) lined by the endothelium at the peritumoral area, not intratumoral^15^. The LVI was graded as follows: 1 (absent, not identified of LVI); 2 (mild, identified a few foci of LVI, but not easy to define); 3 (moderate, LVI present, easy to define); 4 (marked, frequent LVI, or occasionally present, but identified within the large muscularized vessels). Vessel ectasia was defined as congestive or dilated vascular channels, often filled with proteinaceous fluid within open-lumina vessels, which was considered as a histopathological feature of the hemodynamic change related to mechanical obstruction of lymphatic or blood flow and graded as follows: 1 (absent, not identified); 2 (mild, a few foci of vessel ectasia identified, but not easy to define); 3 (moderate, present, easy to define); 4 (marked, frequently present, or identified in the large muscularized vessels). Stromal fibrosis was defined as an intratumoral and in-proximity peritumoral stromal change with a variable degree of collagenous matrix deposition^16^, and was graded as follows: 1 (absent, not identified collagenous deposition); 2 (mild, focally identified, but less than 10% of the entire tumor area in cases of spatially uneven collagen lay-down); 3 (moderate, easily identified or 10–50% of the entire tumor area); 4 (marked, diffusely identified or more than 50% of the entire tumor area). Growth pattern was categorized as 1 (expanding, showing only the pushing border), and 2–4 (infiltrative). The infiltrative scheme was graded as follows: 2 (mild, focally infiltrative, but not easy to define); 3 (moderate, focally infiltrative, easy to define); 4 (marked, diffusely infiltrative). The representative images for each grade of LVI, vessel ectasia, stromal fibrosis, and growth pattern are presented in Appendix E1 (online).

Tumor budding was defined as single cells or clusters of less than 5 cells at the leading edge of tumor invasion based on the International Tumor Budding Consensus Conference^17^. Tumor budding was assessed for a hotspot count on a 20 × objective lens magnification and was graded as follows: 1 (absent, not identified); 2 (low, < 4 buds); 3 (intermediate, 5–9 buds); 4 (high, ≥ 10 buds). Tumor necrosis was defined as the coagulative necrosis of tumor cells of the invasive carcinoma component and was graded as follows in a percent scale: 1 (absent); 2 (≤ 10%); 3 (≤ 20%); 4 (> 20%). Tumor infiltrating lymphocytes (TILs) were defined as mononuclear immune cell infiltrates (i.e., lymphocytes and plasma cells) at the in-proximity peritumoral stroma and was assessed by using the guidelines of the international TIL working group^18^. TIL was measured using a percent scale of mononuclear immune cells in the stromal tissue at the leading edge of the invasive tumor and graded as follows: 1(≤ 10%), 2 (≤ 20%), 3 (≤ 30%), 4 (≤ 40%), 5 (> 40%). Representative TIL was estimated using the average TIL count, not a hotspot. TILs outside the invasive border and around ductal carcinoma in situ, normal lobules, and areas of tumor cell necrosis were not included in the TIL evaluation.

### Statistical analysis

Comparisons of characteristics between patients with and without PE were performed using independent *t* test (age), Chi-squared test (histologic type, HR status, HER2 status, Ki-67 index, and subtype) or the Chi-squared test for trend (T stage, N stage, histologic grade, and detailed histopathological characteristics). Odds ratios (ORs) with 95% confidence interval for PE were calculated for each characteristic using univariate logistic regression. Adjusted ORs were calculated for detailed histopathological characteristics using multivariate logistic regression after an adjustment of baseline clinicopathological characteristics (age, T stage, N state, histologic grade, and subtype with *P* < 0.05 in univariate analyses) to evaluate an independent association of detailed histopathological characteristics; HR status, HER2 status, Ki-6 index were excluded from the multivariate analyses due to multicollinearity with subtype. Due to our semi-quantitative, tiered grading system (1–4 or 5) for detailed histopathological characteristics, ORs were calculated as continuous variables. All statistical analyses were performed with the MedCalc statistical software, version 17.1 (Mariakerke, Belgium). Two-tailed *P* values less than 0.05 were considered statistically significant.

## Results

Table 1 describes the baseline characteristics of the 136 patients and associations with PE. Among them, 34 patients (25.0%) had peritumoral edema at preoperative breast MRI. The mean age at the initial diagnosis was 49 ± 9 years (standard deviation), which was higher in the patients with PE than those without PE (53.7 years vs. 47.7 years, *P* < 0.001). PE was more frequently found in patients with higher pathologic T stage (*P* < 0.001), higher pathologic N stage (*P* = 0.004), higher histologic grade (*P* < 0.001), negative HR status (*P* = 0.04), and higher Ki-67 index (*P* < 0.001). Positive HER2 status was more frequently found in patient with PE than those without PE, albeit borderline significance (*P* = 0.05). PE was found in a higher proportion in more aggressive subtypes (luminal B, triple-negative, and HER2-enriched) than luminal A subtype (*P* < 0.001).

**Table 1:** Baseline clinicopathological characteristics associated with peritumoral edema.

Characteristics	All patients	Patients without peritumoral edema (n = 102)	Patients with peritumoral edema (n = 34)	*P* value
Age*, years	49.2 (8.9)	47.7 (8.2)	53.7 (9.4)	< 0.001^a^
**T stage**				< 0.001^b^
T1	86 (63.2)	80 (78.4)	6 (17.6)	
T2	47 (34.6)	20 (19.6)	27 (79.4)	
T3	3 (2.2)	2 (2.0)	1 (2.9)	
**N stage**				0.004^b^
N0	98 (72.1)	79 (77.5)	19 (55.9)	
N1	29 (21.3)	19 (18.6)	10 (29.4)	
N2	8 (5.9)	4 (3.9)	4 (11.8)	
N3	1 (0.7)	0 (0.0)	1 (2.9)	
**Histologic type**				0.83^c^
Ductal	125 (91.9)	94 (92.2)	31 (91.2)	
Lobular	4 (2.9)	3 (2.9)	1 (2.9)	
Others**	7 (5.1)	5 (4.9)	2 (5.9)	
**Histologic grade**				< 0.001^b^
Low	20 (14.7)	20 (19.6)	0 (0.0)	
Moderate	99 (72.8)	76 (74.5)	23 (67.6)	
High	17 (12.5)	6 (5.9)	11 (32.4)	
**HR status**				0.04^c^
Negative	26 (19.1)	15 (14.7)	11 (32.4)	
Positive	110 (80.9)	87 (85.3)	23 (67.6)	
**HER2 status**				0.05^c^
Negative	106 (77.9)	84 (82.4)	22 (64.7)	
Positive	30 (22.1)	18 (17.6)	12 (35.3)	
**Ki-67 index**				< 0.001^c^
< 14%	68 (50.0)	62 (60.8)	6 (17.6)	
≥ 14%	68 (50.0)	40 (39.2)	28 (82.4)	
**Subtype**				< 0.001^c^
Luminal A	56 (41.2)	52 (51.0)	4 (11.8)	
Luminal B	54 (39.7)	35 (34.3)	19 (55.9)	
Triple-negative	14 (10.3)	9 (8.8)	5 (14.7)	
HER2-enriched	12 (8.8)	6 (5.9)	6 (17.6)	

Unless otherwise indicated, data are presented as numbers of patients, with percentages in parentheses.

*Data are presented as mean values, with standard deviation in parentheses.

*HR* hormone receptor, *HER2* human epidermal growth factor receptor 2.

**Others include mucinous (n = 4) and metaplastic (n = 3) carcinomas.

^a^Independent *t* test, ^b^Chi-squared test for trend, ^c^Chi-squared test.

**Figure 2:**
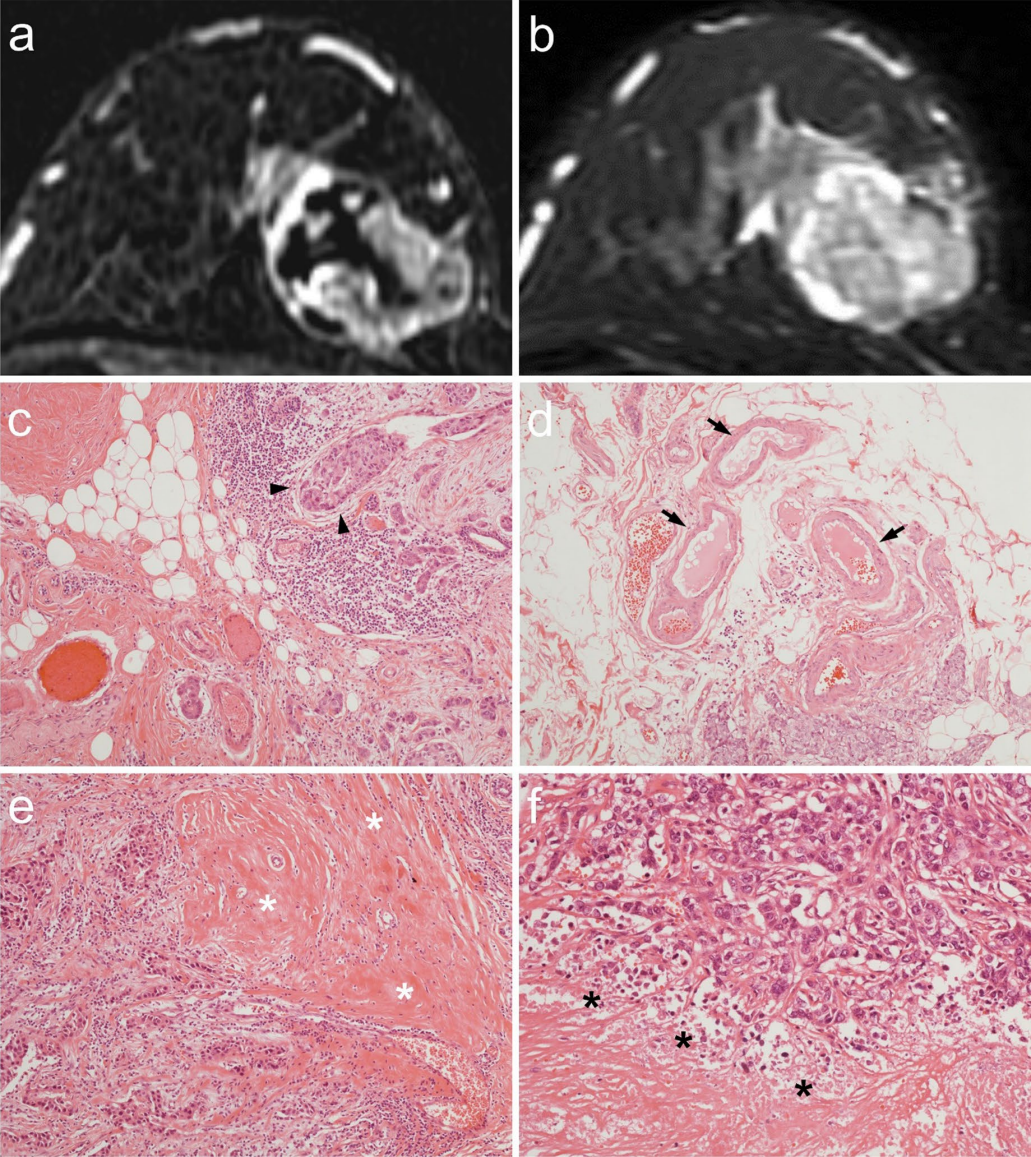
Preoperative breast MR images in a 54-year-old woman with invasive ductal carcinoma. (**a**) Fat-suppressed T1-weighted axial contrast-enhanced images shows a 4 cm mass in the left breast. (**b**) Fat-suppressed T2-weighted axial image shows peritumoral edema seen as high signal intensity surrounding the tumor (arrows). Microscopic pictures demonstrated a moderate grade of lymphovascular invasion (**c**, indicated by black arrowheads), a moderate grade of vessel ectasia (**d**, indicated by black arrows), a marked grade of stromal fibrosis (**e**, indicated by white asterisks), and a ≥ 30% of tumor necrosis (**f**, indicated by black asterisks) (hematoxylin–eosin stain; original magnification, **c**–**e** × 100; and **f** × 200).

Detailed histopathological characteristics of the patients with PE and those without PE are summarized in Table 2. PE was associated with a greater degree of lymphovascular invasion (*P* < 0.001, Fig. 2); marked degree of LVI was found in 44.1% (15/34) of the patients with PE as compared to 17.6% (18/102) in those without PE. PE was associated with a greater degree of vessel ectasia (*P* < 0.001); marked degree of vessel ectasia was found in 64.7% (22/34) of the patients with PE as compared to 20.6% (21/102) in those without PE. PE was associated with a greater degree of stromal fibrosis (*P* = 0.003); moderate or marked degree of stromal fibrosis was found in 32.4% (11/34) of the patients with PE as compared to 10.8% (11/102) in those without PE. PE was associated with less degree of infiltrative growth pattern (*P* < 0.001); 47.1% (16/34) and 41.2% (14/34) of the patients with PE have moderate and marked infiltrative growth pattern, respectively, in comparison to the majority (82.4%, 84/102) of the patients without PE having marked infiltrative growth pattern. PE was associated with a greater degree of tumor necrosis (*P* < 0.001); 50.0% (17/34) of patients with PE had greater than 10% of tumor necrosis as compared to the majority (93.1%, 95/102) of the patients without PE had no tumor necrosis. Tumor budding and tumor infiltrative lymphocytes were not associated with PE (*P* = 0.72 and *P* = 0.16, respectively).

**Table 2:** Detailed histopathological characteristics associated with peritumoral edema.

Characteristics	All patients	Patients without peritumoral edema (n = 102)	Patients with peritumoral edema (n = 34)	*P* value*
**Lymphovascular invasion**				< 0.001
1 (Absent)	29 (21.3)	28 (27.5)	1 (2.9)	
2 (Mild)	24 (17.6)	18 (17.6)	6 (17.6)	
3 (Moderate)	50 (36.8)	38 (37.3)	12 (35.3)	
4 (Marked)	33 (24.3)	18 (17.6)	15 (44.1)	
**Vessel ectasia**				< 0.001
1 (Absent)	2 (1.5)	2 (2.0)	0 (0.0)	
2 (Mild)	23 (16.9)	21 (20.6)	2 (5.9)	
3 (Moderate)	68 (5.0)	58 (56.9)	10 (29.4)	
4 (Marked)	43 (31.6)	21 (20.6)	22 (64.7)	
**Stromal fibrosis**				0.003
1 (Absent)	60 (44.1)	50 (49.0)	10 (29.4)	
2 (Mild)	54 (39.7)	41 (40.2)	13 (38.2)	
3 (Moderate)	21 (15.4)	11 (10.8)	10 (29.4)	
4 (Marked)	1 (0.7)	0 (0.0)	1 (2.9)	
**Growth pattern**				< 0.001
1 (Expanding)	4 (2.9)	3 (2.9)	1 (2.9)	
2 (Infiltrative, mild)	4 (2.9)	1 (1.1)	3 (8.8)	
3 (Infiltrative, moderate)	30 (22.1)	14 (13.7)	16 (47.1)	
4 (Infiltrative, marked)	98 (72.1)	84 (82.4)	14 (41.2)	
**Tumor necrosis**				< 0.001
1 (Absent)	106 (77.9)	95 (93.1)	11 (32.4)	
2 (≤ 10%)	8 (5.9)	2 (2.0)	6 (17.6)	
3 (≤ 20%)	20 (14.7)	5 (4.9)	15 (44.1)	
4 (> 20%)	2 (1.5)	0 (0.0)	2 (5.9)	
**Tumor budding**				0.72
1 (Absent)	11 (8.1)	10 (9.8)	1 (2.9)	
2 (Low)	24 (17.6)	17 (16.7)	7 (20.6)	
3 (Intermediate)	40 (29.4)	25 (24.5)	15 (44.1)	
4 (High)	61 (4.9)	50 (49.0)	11 (32.4)	
**Tumor infiltrating lymphocytes**				0.16
1 (≤ 10%)	93 (68.4)	76 (74.5)	17 (50.0)	
2 (≤ 20%)	19 (14.0)	10 (9.8)	9 (26.5)	
3 (≤ 30%)	15 (11.0)	9 (8.8)	6 (17.6)	
4 (≤ 40%)	3 (2.2)	2 (2.0)	1 (2.9)	
5 (> 40%)	6 (4.4)	5 (4.9)	1 (2.9)	

Data are presented as numbers of patients, with percentages in parentheses.

*Chi-squared test for trend.

Univariable analysis (Table 3) showed that characteristics associated with PE were age (*P* = 0.001), T stage (*P* < 0.001), N stage (*P* = 0.01), histologic grade (*P* < 0.001), HR status (*P* = 0.03), HER2 status (*P* = 0.03), Ki-67 index (*P* < 0.001), subtype (*P* < 0.001), lymphovascular invasion (*P* < 0.001), vessel ectasia (*P* < 0.001), stromal fibrosis (*P* = 0.004), growth pattern (*P* = 0.001), and tumor necrosis (*P* < 0.001). After adjustments of baseline clinicopathological characteristics that were significantly associated with PE, LVI (*P* = 0.009), vessel ectasia (*P* = 0.021), stromal fibrosis (*P* = 0.024), growth pattern (*P* = 0.036), and tumor necrosis (*P* < 0.001) were also associated with PE. In comparison with patients without PE, patients with PE were more likely to have a higher degree of lymphovascular invasion (OR, 2.9), vessel ectasia (OR, 3.3), stromal fibrosis (OR, 2.5), lesser degree of infiltrative growth pattern (OR, 0.4), and higher portion of tumor necrosis (OR, 1.4).

**Table 3:** Logistic regression analyses of detailed histopathological characteristics associated with peritumoral edema.

Characteristics	Unadjusted		Adjusted*	
	Odds ratio^1^	*P* value^1^	Odds ratio^2^	*P* value^2^
Lymphovascular invasion	2.2 (1.4, 3.4)	< 0.001	2.9 (1.3. 6.4)	0.009
Vessel ectasia	4.5 (2.2, 9.3)	< 0.001	3.3 (1.2, 8.9)	0.021
Stromal fibrosis	2.2 (1.3, 3.7)	0.004	2.5 (1.1, 5.6)	0.024
Growth pattern	0.4 (0.2, 0.7)	0.001	0.4 (0.2. 0.9)	0.036
Tumor necrosis	1.4 (1.3, 1.6)	< 0.001	1.4 (1.1, 1.7)	< 0.001
Tumor budding			0.8 (0.4, 1.7)	0.579
Tumor infiltrating lymphocytes			1.0 (0.9, 1.1)	0.926

*Adjusted for the clinicopathological characteristics significantly associated with peritumoral edema univariate analysis (age, T stage, N stage, histologic grade, and subtype); hormone receptor status, human epidermal growth factor receptor 2 status, and Ki-67 index were excluded due to the multicollinearity with subtype.

Due to our semi-quantitative, tiered grading system (1–4 or 5) for detailed histopathological characteristics, ORs were calculated as continuous variables. Odds ratio^1^ and *P* value^1^ for unadjusted logistic regression analyses. Odds ratio^2^ and *P* value^2^ for adjusted logistic regression analyses.

## Discussion

In our study, we tried to find detailed histopathological characteristics, as well as baseline clinicopathological characteristics that are associated with peritumoral edema seen at breast MRI in patients with breast cancers. This is the first interpretative histopathological analyses to elaborate on the putative mechanism of the peritumoral edema, as an emerging semantic feature of breast MRI. Detailed histopathological findings of LVI (*P* = 0.009), vessel ectasia (*P* = 0.021), stromal fibrosis (*P* = 0.024), growth pattern (*P* = 0.036), and tumor necrosis (*P* < 0.001) that were associated with PE will improve our understanding on the tumor microenvironment of local physical properties, tumor hypoxia, and extracellular matrix.

One of the major findings delineated in this study is that LVI was more commonly observed in breast cancers with PE than in those without PE (97.1% [33/34] vs. 74.2% [69/93]). These results were concordant with previous studies by Gemici et al. and Cheon et al., where they also found a higher proportion of the presence of LVI in patients with PE than in those without PE (50–56% vs. 11–26%)^9,3^. However, the LVI was not significantly associated with PE in the other previous study^2^. We assume that these discrepancies came from the difference in the study design. For this study, we retrospectively reviewed LVI using quantitative grading system with careful inspection and included patients with clear PE for comparison, while the LVI in the pathologic report was used and patients with equivocal PE were excluded in the previous study.

Additionally, the microscopic finding of vessel ectasia was also more frequently found in breast cancers with PE, probably due to a secondary hemodynamic change related to mechanical obstruction of lymphatic or blood flow by tumor emboli with LVI or a mass effect of the tumor itself. We believe that a major microscopic mechanism underlying PE is the mechanical obstruction of the local lymphovascular system and upcoming fluid retention or leakage in the peritumoral space, rather than inflammatory cell infiltration, among different speculations over PE that have been proposed in prior studies^4,7^. A positive association between PE and tumor size consistently seen in our results and previous studies may support the speculation that the mechanical obstruction has an important role to play in the phenomenon of PE, since the large space-occupying tumors are apt to induce a mass-effect and afterward mechanical obstruction surrounding the tumor.

We have noted that PE was independently associated with the higher degree of stromal fibrosis, possibly suggesting a state of increased deposition, or altered organization of extracellular matrix (ECM). ECM is one of the critical components of tumor microenvironment and provides the structural foundation and mechanical integrity for tissue function^19^. PE with a higher degree of stromal fibrosis may reflect the physical properties of the tumor microenvironment, specifically a higher degree of mechanical stress (increased ECM stiffness) which is related to tumor cell invasion as well as metastatic dissemination^20,21^. This understanding may be helpful in elaborating the poor prognostic value of the presence of PE. Furthermore, the higher stromal fibrosis associated with PE seems to be also responsible for the local physical properties of mechanical obstruction because that the higher stromal fibrosis with abundant collagen lay-down may induce mechanically rigid tumor microenvironment.

Another noteworthy feature was that tumor necrosis was significantly associated with PE. In general, tumor necrosis is caused by tissue hypoxia, wherein tumor cells induce neoangiogenesis to overcome the hypoxic conditions and increased demands of oxygen and nutrient supply^22^. Such newly formed vessels are not robust because of the incomplete vascular structure and abnormal lymphatic drainage, leading to a vascular leakage^23^ and an increase in interstitial fluid pressure^24^; thus, PE can be a phenomenological feature that is related to the hypoxic condition of the tumor, in addition to mechanical obstruction of local lymphovascular and interstitial flow system. Meanwhile, tumor hypoxia affects the biology of tumors such as apoptosis repression, pro-survival gene expression, and epithelial to mesenchymal transition^25,26^, which consequently amplifies the tumor aggressiveness and therapeutic resistance^27,28^. Of note, the tumor hypoxia is one of the key factors related to the poor prognosis, for example, hypoxia-inducible factor-1α is known to be involved in tumor growth, local invasion, extravasation at sites of metastasis, and maintenance of the cancer stem cell phenotype^29^; moreover, studies have determined that poor tumor oxygenation is the strongest prognostic indicator of radiotherapy treatment outcome^30,31^. These biological findings seem to be line with the results in our study as well as prior studies, wherein PE was related to the aggressive biological profiles of tumors and higher rate of distant metastases in patients with breast cancer^1,2,32,33^.

As a potential imaging biomarker for the comprehensive risk classification and prognostication for the patients with breast cancer, PE may be of value in defining the appropriate therapeutic targets toward patients who need a more intensive adjuvant management. Notably, our semi-quantitative analysis of the histopathological interpretation related to the radiological feature could provide the explainable and practical medical image information and ensure the use of the more integrative information for patient management. However, this study had several limitations. First, the assessment of PE on breast MRI was subjective despite repeated verification and consensus. Second, the histopathological assessment using a semi-quantitative scale may be less validated. Third, the correlation between histopathological quantification scheme and radiological image identification was only partially figured out. Further research on these issues can provide a novel understanding of pathophysiologic mechanism and potential relationship between the other components of the tumor microenvironment.

In summary, detailed histopathological characteristics of lymphovascular invasion, stromal fibrosis, and tumor necrosis as well as baseline clinicopathological characteristics of age and histologic grade were associated with peritumoral edema at breast MRI. Our findings indicate that breast MRI with an assessment of peritumoral edema is able to give an insight as regards local tumor microenvironment; nonetheless, additional studies are needed to fully understand the mechanisms underlying peritumoral edema.

## Supplementary Information

Supplementary Figures.

## Abbreviations

MRI: Magnetic resonance imaging
PE: Peritumoral edema
OR: Odds ratio
ER: Estrogen receptor
PR: Progesterone receptor
HER2: Human epidermal growth factor receptor 2
HR: Hormone receptor
H&E: Hematoxylin and eosin
LVI: Lymphovascular invasion
TIL: Tumor infiltrating lymphocyte
