# Supplementary material for: Peritumoral edema in breast cancer at preoperative MRI: an interpretative study with histopathological review toward understanding tumor microenvironment
Source: Sci Rep. 2021 Jun 21;11:12992. doi: 10.1038/s41598-021-92283-z (PMC8217499; doi:10.1038/s41598-021-92283-z)

**Supplementary Figure 1. Semi-quantitative histopathological assessment for lymphovascular invasion.** The lymphovascular invasion was evaluated according to the four-tiered scales: (A) absent, not identified of LVI; (B) mild, identified a few foci of LVI, but not easy to define; (C) moderate, LVI present, easy to define; and, (D) marked, frequent LVI (H&E stain; A, C, and D, x20; B, x40; the lymphovascular emboli were indicated by black arrowheads).


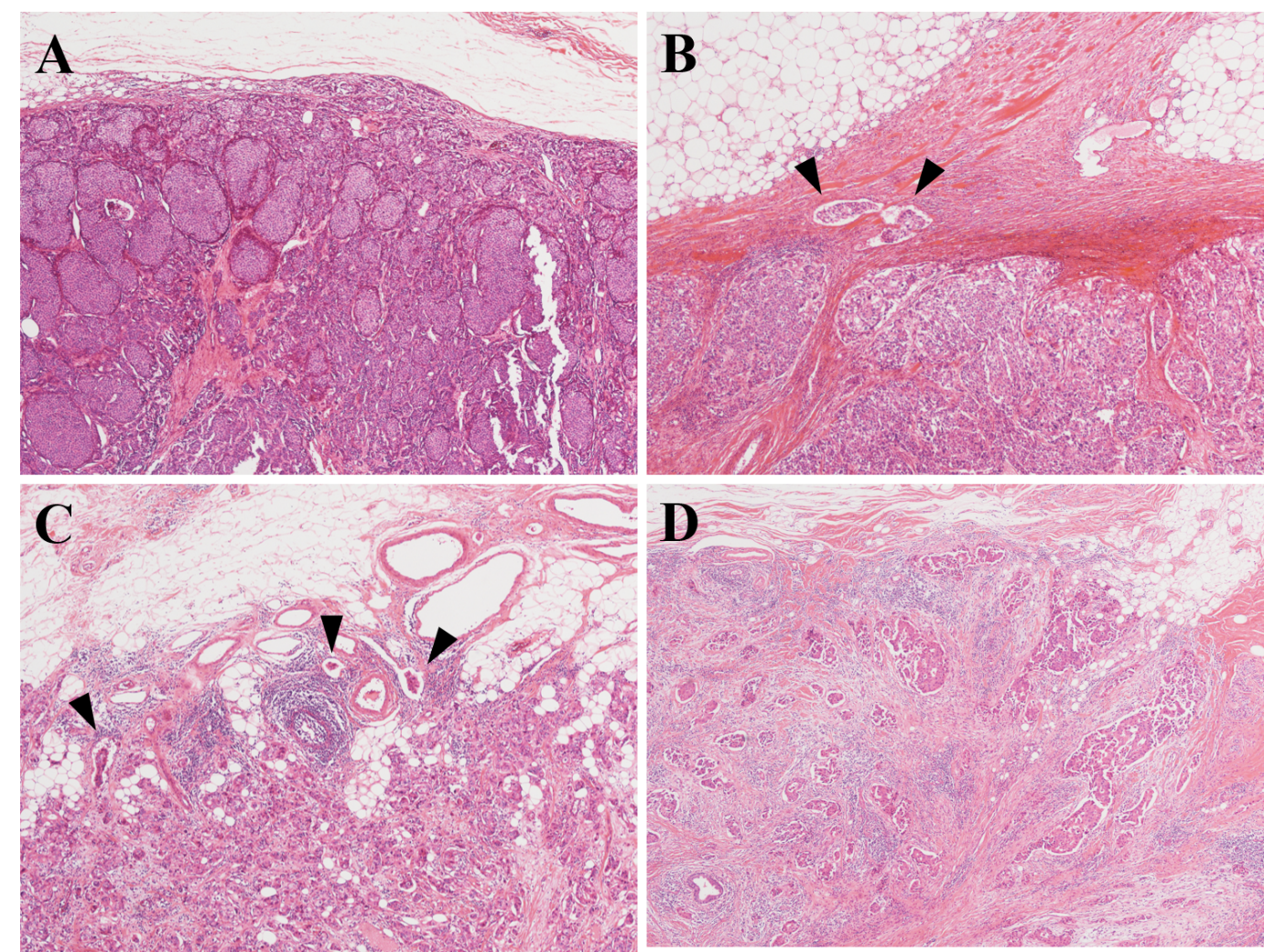


**Supplementary Figure 2. Semi-quantitative histopathological assessment for vessel ectasia.** The vessel ectasia was graded as follows: (A) absent, not identified; (B) mild, a few foci of vessel ectasia identified, but not easy to define; (C) moderate, present, easy to define; and (D) marked, frequently present (H&E stain; A and B x40; C and D, x20; indicated by black arrowheads).


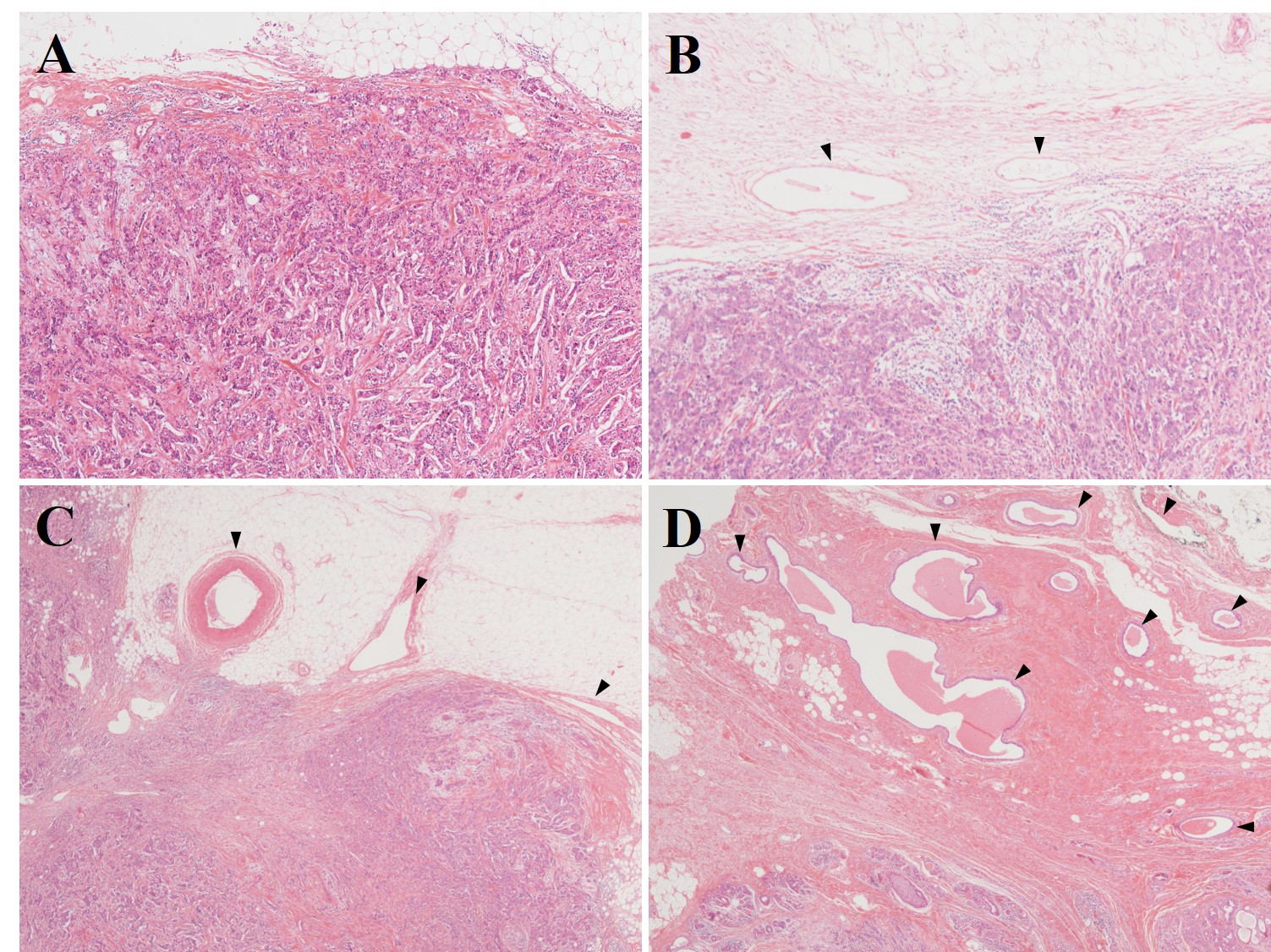


**Supplementary Figure 3. Semi-quantitative histopathological assessment for stromal fibrosis.** The stromal fibrosis was analyzed according to the following schemes: (A) absent, not identified collagenous deposition; (B) mild, focally identified, but less than 10% of the entire tumor area; (C) moderate, easily identified; and (D) marked, diffusely identified collagen deposition, more than 50% of the entire tumor area (H&E stain; A, x20; B and D, x40; C, x100; indicated by black dotted-lines and asterisks).


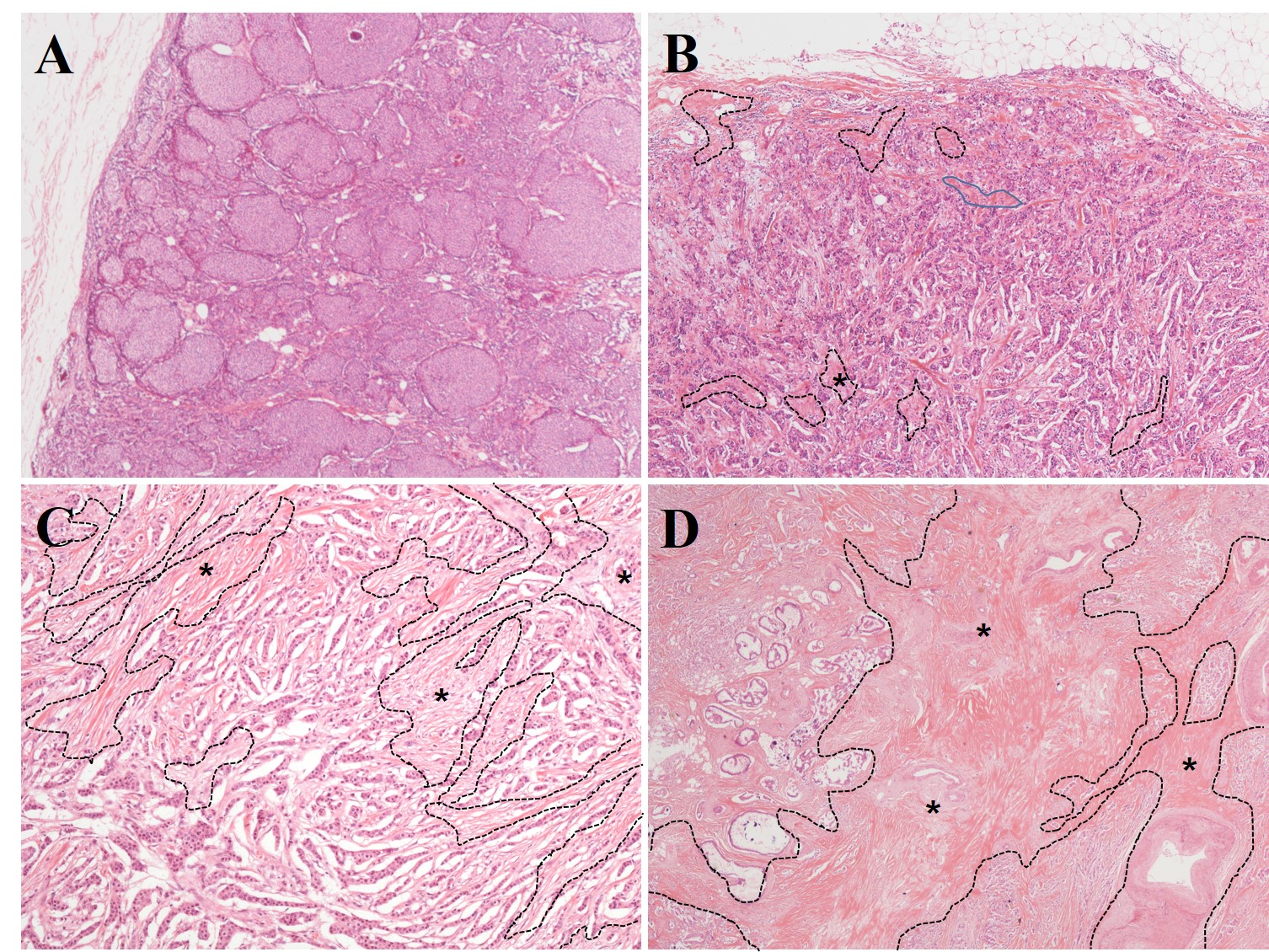


**Supplementary Figure 4.** . **Semi-quantitative histopathological assessment for growth pattern.** The growth pattern was grouped as expanding, showing only pushing border (A), and infiltrative (B to D). The infiltrative degree was evaluated as follows: (B), focally infiltrative, but not easy to define; (C) moderate, focally infiltrative, easy to define; and, (D) marked, diffusely infiltrative (H&E stain; A to D, x20; the microscopic foci of infiltrative growth showing mild degree were indicated by black arrowheads).


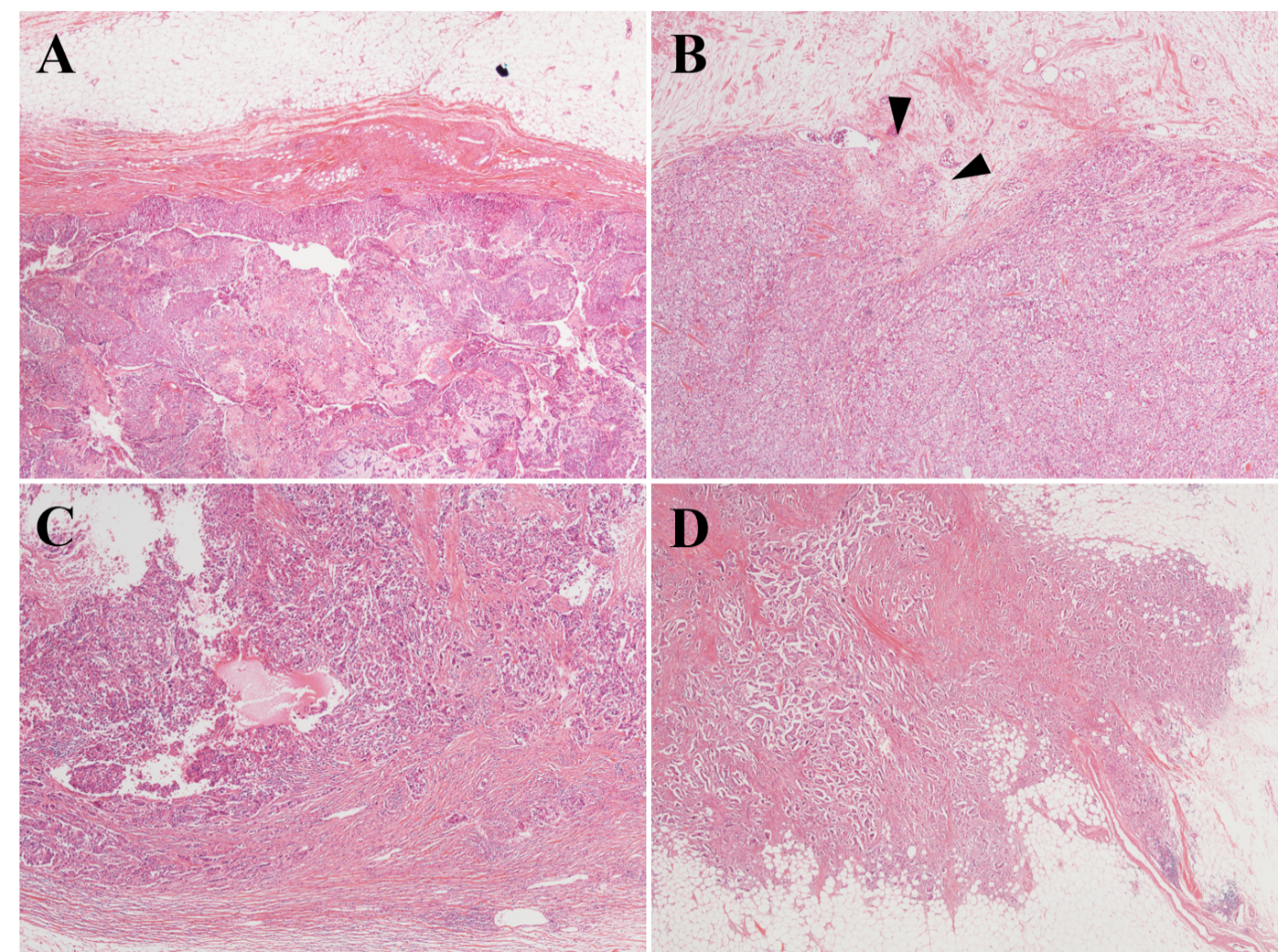

Supplement: Supplementary file 1 — Supplementary Figures. [file 41598_2021_92283_MOESM1_ESM.docx]
